# Online learning modules improve confidence in providing gender affirming care for youth

**DOI:** 10.1186/s12909-024-06517-5

**Published:** 2024-12-19

**Authors:** Juanita K. Hodax, Nicole F. Kahn, Julia M. Crouch, Janis L. Sethness, Kevin M. Bocek, Catherine Sumerwell, Gina M. Sequeira

**Affiliations:** 1 Division of Endocrinology, Department of Pediatrics, Seattle Children’s Hospital, 4800 Sand Point Way NE, MS OC.7.920, Seattle, WA 98105 USA; 2 Division of Adolescent Medicine, Department of Pediatrics, Seattle Children’s Hospital, Seattle, WA USA

**Keywords:** Education, Gender affirming care, Online learning

## Abstract

**Purpose:**

Healthcare providers often lack training and education in caring for gender diverse youth. We aimed to explore changes in provider confidence and behaviors following the implementation of an online learning course focused on gender affirming care for youth.

**Methods:**

An asynchronous, online training consisting of 2 modules was made available in October 2021. Participants were asked to complete 3 surveys for each module they completed: a pre-survey, a post-survey, and a 3-month follow-up survey. Surveys included demographic data and an assessment of provider confidence and self-reported behaviors related to the provision of gender affirming care for adolescents that were in line with the objectives outlined in each module. Paired sample t-tests were used to compare participant confidence at all timepoints.

**Results:**

Participants (*n* = 487) completed at least one survey from one module. There was notable diversity in provider types, including mental health providers (*n* = 86, 17.7%), community health workers (*n* = 71, 14.6%), and medical students or health professionals (*n* = 61, 12.5%). Participants were significantly more confident in all aspects of care provision when comparing pre-surveys and post-surveys (*p* < 0.001 for all survey items focused on change in provider confidence ). At 3-month follow-up after module completion, improvements in confidence were sustained in providing information and resources to adolescents and their families, and having conversations with them about gender identity along with the importance of using affirming language. However, these modules did not result in self-reported behavior change regarding provision of gender affirming hormones or puberty blockers.

**Conclusions:**

Online learning modules may be an effective means of educating a large number of healthcare providers about caring for gender diverse youth in a variety of settings and across disciplines.

**Supplementary Information:**

The online version contains supplementary material available at 10.1186/s12909-024-06517-5.

## Introduction

Gender diverse youth are disproportionately burdened by poor health outcomes, including higher rates of depression, anxiety, suicidality, self-harm, and eating disorders compared to their cisgender peers [1–6]. They are also more likely to experience societal discrimination resulting in economic marginalization, social isolation, and trauma [6–8]. These conditions put them at higher risk for substance use, violence, homelessness, human immunodeficiency virus, and other sexually transmitted infections [7, 9, 10]. Access to gender affirming medical care such as puberty blockers, hormone therapy, and gender affirming surgeries during adolescence and adulthood has been shown to improve psychosocial functioning in gender diverse people [2, 11–16].

Unfortunately, there are numerous barriers to gender diverse young people receiving gender affirming care, including financial concerns, discrimination, geographic limitations, health systems issues, and socioeconomic barriers [17–20]. The biggest barrier to receiving health care reported by gender diverse individuals is a lack of access due to the limited number of providers who are sufficiently knowledgeable about transgender health [17, 18]. Provider knowledge is lacking due in large part to the lack of education in transgender health offered by medical and other healthcare professions schools. In fact, providers across specialties express that they do not have the training or experience necessary to care for gender diverse populations [21–33]. Because transgender care for adolescents is frequently provided using a multidisciplinary approach, access to gender care education across disciplines is critically important [21].

Improving healthcare access and ensuring affirming clinical environments for transgender populations requires that training in the care of gender diverse populations be available for all members of the healthcare workforce [34]. Patients may encounter schedulers, insurance coordinators, administrative staff, medical assistants, and other healthcare personnel before they ever enter an exam room. Several interventions aimed at improving transgender health knowledge have been implemented among health professional students and trainees. Studies have demonstrated that education via didactics or direct patient care can be effective in improving knowledge, attitudes, preparedness, comfort, and willingness to provide care for gender diverse people [21, 35–38]. Few studies have specifically examined the use of online modules to enhance medical providers’ knowledge of gender affirming care [39–41]. One investigation offered a transgender youth curriculum via online modules to medical trainees and found this was effective at improving learners’ perceived knowledge of transgender youth health concerns and enhanced transgender-related health training [40].

We created a new online learning course focused on gender affirming care provision for adolescents that was available to any interested learner, with the target audience being healthcare providers. Unlike prior studies, these modules were widely used by mental health providers and clinic staff, as well as all medical providers in training and already in practice [39–41]. We aimed to explore change in provider confidence and self-reported behaviors following the completion of our online learning module using a survey assessment tool.

## Methods

### Online learning module

A group of adolescent gender care providers created an asynchronous, online learning course to educate healthcare providers about gender affirming care for adolescents [42]. The course was titled “Caring for Gender Diverse Youth”, and consisted of two separate modules “Course One: Creating a Welcoming Environment” and “Course Two: Gender Affirming Medical Care”. The modules were interactive, with patient scenarios intermixed with educational content. The learning objectives and outline of the two modules are available in Table 1. Educational content was developed based on guidelines from the World Professional Association of Transgender Health, the Endocrine Society, and University of California San Francisco [43–45]. Both learning modules are available for free online and can be accessed through the Seattle Children’s Gender Clinic website. The intended audience was anyone in the healthcare field. Continuing medical education credit was offered to participants who completed the modules.

**Table 1:** Online Learning Module Objectives and Outline

Module 1: Creating a Welcoming Environment	Module 2: Gender Affirming Medical Care
**Objectives** • Define key terminology and concepts important in the care of the gender diverse youth • Discuss the health disparities and protective factors associated with gender diverse youth • Describe at least three strategies for creating an affirming clinical environment for gender diverse patients	**Objectives** • Explain medical options for care in pubertal and post-pubertal gender diverse youth • Compare dosing regimen options for hormone treatment • Identify options for surgical care
**Outline** • Background terms and definitions • Health and safety considerations • Protective factors • Settings for gender affirming medical care • Creating a welcoming environment	**Outline** • Beginning gender affirming medical care: Initial conversations and assessments • Overview of care options • Pubertal suppression • Gender affirming hormones • More considerations: Surgical options, Nonbinary patients, and referrals

Any individual who accessed one of the modules was given the opportunity to consent to participate in a research study approved by the Seattle Children’s Institutional Review Board (STUDY00003290). While the intended audience of the learning modules was healthcare providers, the module was available online for free to anyone who chose to complete it. There was not any specific advertising for the module or the research study, though the link to the module was shared during other educational sessions related to gender-affirming care. Those who did not consent to the research study could access the learning module without participating in the study.

### Module evaluation

Participants who consented to participate were asked to answer 3 surveys for each module they completed: a pre-survey before starting the module, a post-survey immediately after finishing the module, and a follow-up survey that was emailed 3 months after the module was completed. Surveys completed between October 2021 and February 2023 were included in the analysis.

Surveys for each module included items exploring providers’ demographic characteristics such as practice location, provider type, years in practice, number of gender diverse youth seen. Appendix 1 shows the full list of survey questions. Some survey items assessing provider confidence and self-reported behaviors were adapted from the Trans-Inclusive Provider Scale (TIPS), a previously validated 27-item Likert Scale measure to assess provider’s inclusivity toward transgender and gender diverse adults [46]. The Likert Scale options were on a scale of 1 to 5, with 1 “This is not true at all”, 2 “This is rarely true”, 3 “This is somewhat true”, 4 “This is usually true”, 5 “This is always true” [46]. To ensure items were inclusive of aspects of gender-affirming care provision that were unique to the needs of youth, adaptations to the TIPS scale were made by the multidisciplinary study team prior to implementation, including both adjustments to the language of individual items and removal of specific items deemed not relevant or appropriate to care provision for youth. The most notable adjustment to the TIPS scale included the expansion of items to better understand pediatric provide confidence, performing specific aspects of gender-affirming care provision for pediatric patients such as initiating conversations about gender identity with children and their families, and supporting parents in caring for their TGDY, and discussing gender-affirming medical interventions. The same adjustments were made for surveys used in our assessment of alternative educational tools [47].

### Analysis

Participant and practice characteristics were summarized for each module using frequencies and percentages. Next, paired sample t-tests were used to test for change in mean scores on survey items related to participant confidence in their ability to provide various aspects of gender affirming care before completing the module (pre-survey), immediately after completing the module (post-survey), and 3 months after completing the module (3-month follow-up), with a p-level for statistical significance of 0.05. Paired samples t-tests were also used to compare mean scores on self-reported behavior items at pre- and 3-month follow-up only, as behavior would not be possible to change immediately post-completion of the module. All analyses were completed using Stata 14.2 [48].

## Results

### Module 1: “Creating a welcoming environment”

Characteristics of participants who completed Module 1 (*n* = 487) and their practices are shown in Table 2. Participants represented 37 states, Washington DC, Canada, and Mexico, with over one third of participants from Washington state (*n* = 176, 36.1%), and another quarter (*n* = 125, 25.6%) from Texas. A notable number of participants (*n* = 203, 41.7%) were located in a state that was considering or had passed legislation to ban gender affirming care for minors as of the end of the study period [47]. There was great diversity in provider types, the most common of which were mental health providers (*n* = 86, 17.7%), community health workers (*n* = 71, 14.6%), and medical students or health professionals (*n* = 61, 12.5%). Over half had been in practice for ≤ 5 years (*n* = 260, 53.4%), and over 20% had been in practice for 6–10 years (*n* = 105, 21.6%). Most (*n* = 321, 65.9%) reported that patients had access to a mental health provider in their clinic. Finally, just under half (*n* = 220, 45.2%) reported having ≤ 5 gender diverse patients under age 18 on their panel (*n* = 220, 41.1%), with nearly a quarter (*n* = 110, 22.6%) reporting > 15 gender diverse patients under age 18 on their panel.

**Table 2:** Characteristics of participants who completed the baseline (pre) surveys for Modules 1 and 2

*n* (%)	Module 1 (*n* = 487)	Module 2 (*n* = 195)
**U.S. Region**		
West	208 (42.7)	72 (36.9)
Southwest	128 (26.3)	53 (27.2)
Northeast	49 (10.1)	16 (8.2)
Southeast	43 (8.8)	27 (13.8)
Midwest	28 (5.7)	15 (7.7)
Outside U.S.	2 (0.4)	1 (0.5)
Missing	29 (6.0)	11 (5.6)
**Provider type**		
Advanced practice provider (ARNP, PA)	31 (6.4)	18 (9.2)
Medical assistant	54 (11.1)	34 (17.4)
Medical student or health professional	61 (12.5)	8 (4.1)
Mental health provider (psychology, social work)	86 (17.7)	31 (15.9)
Nurse	22 (4.5)	9 (4.6)
Physician (MD, DO)	42 (8.6)	9 (4.6)
Resident (MD, DO)	7 (1.4)	2 (1.0)
Missing	7 (1.4)	3 (1.5)
Other (write in)	177 (36.3)	81 (41.5)
Admin	12 (2.5)	5 (2.6)
Advocate	3 (0.6)	
Care/case coordinator/navigator	24 (4.9)	11 (5.6)
Chaplain/Spiritual	2 (0.4)	1 (0.5)
Community health worker	71 (14.6)	31 (15.9)
Counselor		2 (1.0)
Dietitian	2 (0.4)	2 (1.0)
Educator/Health educator	16 (3.3)	11 (5.6)
Intervention/Treatment specialist	11 (2.3)	5 (2.6)
Other	3 (0.6)	
Other public health/prevention	6 (1.2)	3 (1.5)
Outreach/engagement	6 (1.2)	5 (2.6)
Pharmacist/Tech	4 (0.8)	2 (1.0)
Rehabilitation	3 (0.6)	
Student (other/undefined)	7 (1.4)	1 (0.5)
Youth care worker	4 (0.8)	
**Years in practice**		
≤ 5	260 (53.4)	88 (45.1)
6–10	105 (21.6)	51 (26.2)
11–15	39 (8.0)	15 (7.7)
16–20	30 (6.2)	14 (7.2)
> 20	53 (10.9)	27 (13.8)
**Access to mental health provider in clinic**		
No	106 (21.8)	49 (25.1)
Yes	321 (65.9)	124 (63.6)
Multiple practice locations	4 (0.8)	2 1.59)
N/A	27 (5.5)	5 (2.6)
Missing	22 (4.5)	12 (6.2)
Other	7 (1.4)	3 (1.5)
**Percent of patient panel using Medicaid insurance**		
≤ 25%	103 (21.1)	38 (19.5)
26–50%	110 (22.6)	42 (21.5)
51–75%	140 (28.7)	58 (29.7)
76–100%	103 (21.1)	44 (22.6)
Missing	31 (6.4)	13 (6.7)
**Number of gender diverse patients under age 18**		
≤ 5	220 (45.2)	82 (42.1)
6–10	87 (17.9)	37 (19.0)
11–15	38 (7.8)	15 (7.7)
> 15	110 (22.6)	47 (24.1)
Missing	32 (6.8)	14 (7.2)
**Prescriber** ***(Module 2 only)***		
Yes	-	31 (15.9)
No	-	163 (83.6)
Missing	-	1 (0.5)

Notes: - indicates not applicable, ARNP = advanced registered nurse practitioner, PA = physician assistant, MD = medical doctor, DO = doctor of osteopathic medicine

Results of paired samples t-tests testing for change in confidence from the pre- to post-surveys (*n* = 276–283) and from the pre- to 3-month follow-up surveys (*n* = 19–21, including only the participants that completed the 3-month follow-up surveys) are shown in Table 3.

**Table 3:** Change in confidence from pre- to post-surveys and pre-survey to 3-month follow up after completing Module 1

Pre- to Post-surveys					Pre-survey to 3-month follow up				
*n*	Pre Mean (SD)	Post Mean (SD)	Difference Mean (SD)	t-test Sig.	*n*	Pre Mean (SD)	3 months Mean (SD)	Difference Mean (SD)	t-test Sig.
*I am confident in my ability to…*									
introduce myself to patients with my name and pronouns									
282	3.89 (1.23)	4.23 (0.96)	0.34 (1.26)	t = 4.49 *p* < 0.001*	21	4.19 (0.93)	4.24 (0.83)	0.05 (0.97)	t = 0.22 *p* = 0.41
initiate conversations about gender identity with patients during routine visits									
283	3.71 (1.20)	4.15 (1.00)	0.44 (1.29)	t = 5.74 *p* < 0.001*	21	3.90 (1.18)	4.19 (0.68)	0.29 (1.06)	t = 1.24 *p* = 0.11
support a gender diverse patient in talking with their parent or caregiver about their gender identity									
282	3.88 (1.16)	4.28 (0.95)	0.39 (1.21)	t = 5.48 *p* < 0.001*	21	3.95 (0.92)	4.33 (0.66)	0.38 (0.67)	t = 2.61 *p* = 0.01*
explain to a patient or family the difference between gender identity and sexual orientation									
278	3.97 (1.20)	4.34 (0.97)	0.37 (1.20)	t = 5.16 *p* < 0.001*	21	4.05 (1.12)	4.33 (0.66)	0.29 (0.85)	t = 1.55 *p* = 0.07
explain to a parent or caregiver who is refusing to use a gender diverse patient’s name and pronouns the negative impact this has on mental health (with patient’s consent)									
279	3.82 (1.25)	4.28 (1.01)	0.46 (1.26)	t = 6.07 *p* < 0.001*	21	4.14 (1.15)	4.52 (0.51)	0.38 (0.97)	t = 1.79 *p* = 0.04*
explain to a teacher or school administrator the importance of using a gender diverse patient’s name and pronouns at school (with patient’s consent)									
278	3.80 (1.27)	4.29 (0.99)	0.49 (1.24)	t = 6.61 *p* < 0.001*	21	4.10 (1.18)	4.43 (0.60)	0.33 (1.02)	t = 1.50 *p* = 0.07
provide information to a patient about local, gender-affirming resources youth can access confidentially (i.e., without parental involvement)									
279	3.71 (1.25)	4.16 (1.03)	0.46 (1.19)	t = 6.39 *p* < 0.001*	21	3.76 (1.18)	4.24 (0.70)	0.48 (1.03)	t = 2.12 *p* = 0.02*
provide information to a patient or family about local, gender-affirming resources or supports for parents or caregivers of gender diverse youth									
276	3.69 (1.25)	4.20 (1.01)	0.51 (1.20)	t = 7.05 *p* < 0.001*	21	3.67 (1.24)	4.14 (0.85)	0.48 (0.93)	t = 2.35 *p* = 0.01*

Notes: *p < 0.05, SD = standard deviation, sig = significance

Participants were significantly more confident in their abilities to engage in all of the listed behaviors when comparing the pre- and post- surveys. This change in confidence was sustained at 3-month follow-up surveys in their ability to “support a gender diverse patient in talking with their parent or caregiver about their gender identity” (mean difference: 0.38, t = 2.61, *p* = 0.01), “explain to a parent or caregiver who is refusing to use a gender diverse patient’s name and pronouns the negative impact this has on mental health (with patient’s consent)” (mean difference: 0.38, t = 1.79, *p* = 0.04), “provide information to a patient about local, gender affirming resources youth can access confidentially (i.e., without parental involvement)” (mean difference = 0.48, t = 2.12, *p* = 0.02), and “provide information to a patient or family about local, gender affirming resources or supports for parents or caregivers of gender diverse youth” (mean difference: 0.48, t = 2.35, *p* = 0.01). Changes in self-reported behavior items from the pre- to the 3-month follow-up survey were not statistically significant at the *p* < 0.05 level (Table 4).

**Table 4:** Change in behaviors from pre-survey to 3-month follow up after completing Module 1

*n*	Pre Mean (SD)	3 months Mean (SD)	Difference Mean (SD)	t-test Sig.
Other providers in my practice use patients’ affirmed name and pronouns				
19	4.26 (0.65)	4.16 (1.01)	-0.11 (1.05)	t = -0.44 *p* = 0.67
My office staff use patients’ affirmed name and pronouns				
20	4.35 (0.67)	4.15 (1.04)	-0.20 (1.01)	t = -0.88 *p* = 0.81
I introduce myself to patients with my name and pronouns				
21	3.38 (1.02)	3.38 (1.28)	0.00 (1.34)	t = 0.00 *p* = 0.50
I ask my patients what name and pronouns they use during every clinical encounter				
21	3.29 (1.06)	3.38 (1.16)	0.10 (1.26)	t = 0.35 *p* = 0.37
I use my patient’s affirmed name and pronouns when speaking with them				
21	4.76 (0.44)	4.57 (0.98)	-0.19 (1.08)	t = -0.81 *p* = 0.79
I use my patients’ affirmed name and pronouns when describing them to others				
21	4.67 (0.48)	4.52 (0.98)	-0.14 (1.06)	t = -0.62 *p* = 0.73
I initiate conversations about gender identity with patients during every routine visit				
21	3.00 (1.10)	3.38 (0.97)	0.38 (1.28)	t = 1.36 *p* = 0.09
With their consent, I include my patients affirmed name and pronouns in the EHR				
21	4.48 (0.75)	4.33 (1.24)	-0.14 (1.11)	t = -0.59 *p* = 0.72
With their consent, I include my patients gender identity in the EHR				
21	4.43 (0.75)	4.33 (1.24)	-0.10 (1.14)	t = -0.38 *p* = 0.65

Notes: *p < 0.05, EHR = electronic health record

### Module 2: “Gender affirming medical care”

Characteristics of participants who completed Module 2 (*n* = 195) and their practices were similar to those of Module 1 (Table 2). Participants represented 27 U.S. states and Canada, the largest proportions of whom were from Washington state (*n* = 60, 30.8%) and Texas (*n* = 53, 27.2%). Almost half of participants (*n* = 92, 47.2%) were located in a state that was considering or had passed legislation that bans gender affirming care for minors as of the end of the study period [49]. Many different provider types were represented, most commonly medical providers, including physicians, advanced practice providers, trainees, and students (*n* = 37, 19%), medical assistants (*n* = 34, 17.4%), mental health providers (*n* = 31, 15.9%), and community health workers (*n* = 31, 15.9%). Nearly half had been in practice for ≤ 5 years (*n* = 88, 45.1%), with another quarter having been in practice for 6–10 years (*n* = 51, 26.2%). The majority (*n* = 124, 63.6%) reported that patients in their practice had access to a mental health provider, and the percent of patients on their panels who used Medicaid was relatively evenly distributed across categories, with slightly larger proportions reporting 51–75% (*n* = 58, 29.7%) and 76–100% (*n* = 44, 22.6%). Participants most commonly reported having ≤ 5 gender diverse patients under age 18 on their panel (*n* = 82, 42.1%), followed by > 15 patients (*n* = 47, 24.1%). Finally, 15.9% (*n* = 31) indicated that they were medication prescribers.

Results of paired samples t-tests testing for change in Module 2 participants’ confidence from the pre- to post- surveys (*n* = 144–147) and from the pre- to 3-month follow-up surveys (*n* = 8, including only the participants that completed the 3-month follow-up surveys) are shown in Table 5. Similar to Module 1, participants were significantly more confident in their abilities to engage in all of the listed behaviors when comparing the pre- and post- surveys. However, these were not sustained at 3-month follow-up surveys, and in the case of counseling patients and families about puberty blockers, significantly declined from pre- to 3-month follow-up surveys (mean difference: -0.38, t = 2.05, *p* = 0.04). Among participants who reported that they were licensed to prescribe medications, confidence in their abilities to determine pubertal stage, prescribe, and perform lab monitoring also increased significantly between the pre- and post- surveys (Table 6). The number of responses for the 3-month follow up survey was too small for analyses.

**Table 5:** Change in confidence from pre- to post-surveys and pre-survey to 3-month follow up after completing Module 2

Pre- to Post-surveys					Pre-survey to 3-month follow up				
*n*	Pre Mean (SD)	Post Mean (SD)	Difference Mean (SD)	t-test Sig.	*n*	Pre Mean (SD)	3 months Mean (SD)	Difference Mean (SD)	t-test Sig.
*I am confident in my ability to…*									
counsel patients and families about puberty blockers (e.g., what they do, when they can be initiated, how they are administered, etc.)									
146	2.79 (1.30)	3.51 (1.12)	0.72 (1.14)	t = 7.60 *p* < 0.001*	8	3.38 (1.41)	3.00 (1.20)	-0.38 (0.52)	t = -2.05 *p* = 0.04*
counsel patients and families about hormone therapy like Testosterone and Estradiol (e.g., what it does, when it can be initiated, how it can be administered, etc.)									
146	2.86 (1.33)	3.53 (1.13)	0.67 (1.15)	t = 7.08 *p* < 0.001*	8	3.25 (1.58)	2.75 (1.39)	-0.50 (0.76)	t = -1.87 *p* = 0.05
refer a gender diverse young person under 18 to a local provider who prescribes puberty blockers and gender affirming hormones									
147	3.35 (1.35)	3.76 (1.17)	0.41 (1.20)	t = 4.13 *p* < 0.001*	8	3.50 (1.69)	3.13 1.55	-0.38 (1.41)	t = -0.75 *p* = 0.76
refer a gender diverse young person under 18 for a gender-affirming surgery									
144	3.03 (1.35)	3.67 (1.17)	0.64 (1.25)	t = 6.11 *p* < 0.001*	8	3.25 (1.58)	3.13 (1.55)	-0.13 (1.64)	t = -0.22 *p* = 0.58

Notes: *p < 0.05

**Table 6:** Change in confidence among prescribers from pre- to post-survey after completing Module 2

*n*	Pre Mean (SD)	Post Mean (SD)	Difference Mean (SD)	t-test Sig.
*I am confident in my ability to…*				
determine pubertal stage for a patient who is interested in receiving puberty blockers				
22	3.45 (1.10)	4.45 (0.91)	1.00 (0.98)	t = 4.81 *p* < 0.001*
prescribe medications to suppress menses for gender diverse youth experiencing dysphoria with bleeding				
22	2.91 (1.34)	3.95 (1.25)	1.05 (1.09)	t = 4.50 *p* < 0.001*
prescribe an initial prescription for puberty blockers				
22	2.00 (1.02)	3.27 (1.12)	1.27 (0.94)	t = 6.38 *p* < 0.001*
prescribe a follow up prescription for puberty blockers after another provider prescribed the initial prescription				
21	2.67 (1.35)	4.05 (1.20)	1.38 (1.02)	t = 6.18 *p* < 0.001*
perform lab monitoring for patients receiving puberty blockers				
21	2.33 (1.06)	3.81 (1.12)	1.48 (1.03)	t = 6.56 *p* < 0.001*
prescribe an initial prescription for gender-affirming hormones (Testosterone, Estradiol)				
21	2.33 (1.32)	3.38 (1.28)	1.05 (0.92)	t = 5.21 *p* < 0.001*
prescribe a follow up prescription for gender-affirming hormones (Testosterone, Estradiol) for a patient under age 18 after another provider prescribed the first prescription				
22	2.95 (1.53)	4.00 (1.20)	1.05 (1.05)	t = 4.69 *p* < 0.001*
perform lab monitoring for patients receiving gender-affirming hormones (Testosterone, Estradiol)				
21	2.71 (1.49)	3.95 (1.16)	1.24 (1.09)	t = 5.20 *p* < 0.001*

Notes: *p < 0.05; The number of responses for the 3-month follow up survey was too small for analyses

## Discussion

A large number of participants completed our “Caring for Gender Diverse Youth” online learning course. Thirty-seven states were represented, including several states which have passed or have pending legislation to ban gender affirming medical care for minors. Participants included medical providers, as well as many non-medical providers such as community health workers and caseworkers. The large uptake and diversity of participants suggests that training on gender diverse youth is lacking across many healthcare disciplines, and that providers are interested in receiving additional training in this area.

An interesting finding of our study was the large number of providers completing this training from states where anti-transgender policies were implemented or had legislation pending or passed during the study period [49]. This includes Texas, where 25% of the participants were located, a state that began conducting investigations into families who were affirming of their transgender children during the study period. While we did not do any direct advertisement in Texas, the company hosting the online learning modules has an office based in Texas and provides other online learning modules to community healthcare workers in the area on the same platform our module is posted on. As legislation continues to limit the provision of gender affirming care for adolescents in many states, it will also limit the education and training healthcare providers in those states receive about caring for gender diverse youth. Given these growing limitations, online learning modules and additional educational opportunities for providers are crucial to ensuring all providers learn evidence-based information about caring for gender diverse youth.

In our study, almost 70% of participants who completed the online learning modules were early in their career (less than 10 years post-training), showing that medical education over the past decade is still lacking in this area despite this known need. In undergraduate medical education there is minimal or no inclusion of topics related to transgender health [35, 50], and medical schools in the U.S. and Canada taught a median of only 5 h of LGBTQ-related curricular content [29], leading students to feel underprepared to work with transgender patients [30]. In graduate medical education, one study of family medicine, psychiatry, endocrinology, and urology residents found that residents are largely willing, yet feel unprepared to care for gender diverse patients [32, 33]. Only a small percentage of participants completing the module were medical providers. This may have been due to lack of time for additional education, or lack of interest. Required training in caring for gender diverse people earlier in medical education would likely reach a broader range and larger number of medical providers, and is crucial in ensuring access to healthcare for this vulnerable population.

Evaluation of our online learning course showed that for Module 1 “Creating a Welcoming Environment”, the majority of items asked in our survey of provider confidence and self-reported behaviors were rated fairly high in the pre-survey and improved significantly in the post-survey. The lowest areas of confidence in the pre-survey were regarding providing information about community resources youth can access confidentiality, resources for parents and caregivers, and initiating conversations about gender identity at routine visits. In the 3-month follow-up surveys, improved confidence was retained for many of the areas that involve increasing parental support for youth, including supporting youth in talking with their caregiver about their identity and providing community resources for both youth and their families. Improved confidence in these areas is crucial given the clear impact of improvement in mental health for youth with affirming environments at home and in their communities [2, 11–16, 51, 52].

When looking at 3-month follow-up surveys evaluating provider behaviors, two areas of behavior where improvement was seen from baseline were initiating conversations about gender identity and asking about name and pronouns at routine visits. Though this change was not statistically significant, this improvement suggests asynchronous online learning modules such as ours may be helpful in creating space for youth to talk about their gender identity with trusted adults. However, the lack of statistically significant improvement in self-reported behaviors indicates that participating in one e-learning module may not be sufficient to change provider behaviors, suggesting additional interventions and ongoing support and education in this area may be needed.

In Module 2 “Gender Affirming Medical Care”, though participant’s confidence increased in all areas in the post-survey, 3-month follow-up survey results suggest decreases in provider confidence from baseline with a significant decrease in counseling about puberty blockers. These results are limited due to a very small sample of participants completing the 3-month follow-up survey (only 8 participants), indicating a need for ongoing evaluation of provider behavior change over time. Providers who completed 3-month follow-up were on average more confident at baseline than those who completed pre- and post- surveys. While the small sample of prescribers makes it challenging to determine effects on prescribing practice, the data gathered on other aspects of gender-affirming care remains important and necessary to support gender diverse youth.

Baseline confidence levels for medication prescribers were lowest in providing initial prescriptions for puberty blockers and gender affirming hormones, with higher confidence in ordering laboratory monitoring and even higher confidence in providing ongoing prescriptions for puberty blockers and hormones, as well as providing menstrual suppression. The same pattern was seen in the post-survey, though there was significant improvement in confidence in all areas. These findings suggest that, at least in the short term, it may be more feasible to partner with community providers to support lab monitoring and ongoing prescriptions than for them to prescribe initial prescriptions for gender affirming medications. However, given the increasing demand for services and capacity limitations in pediatric gender clinics, it is also necessary to begin to develop interventions to support community providers in providing initial prescriptions.

### Limitations

Limitations of this study include the low rate of completion of the 3-month follow-up survey, making it challenging to assess long-term changes in confidence and behaviors. There were a variety of healthcare providers who participated, however only few prescribers completed the surveys. In addition, we did not collect detailed demographic information from participants, such as age, gender identity, or sexual orientation. Self-reported assessments of confidence and behavior may have some inherent bias, and we are unable to assess how these changes translate to changes in patient experiences in accessing healthcare.

## Conclusion

Online learning modules may be an effective means of educating a large number of healthcare providers in a variety of settings and disciplines about care for gender diverse youth. We saw significant improvement in confidence immediately after completing the online learning modules. Some areas of improvement in confidence were sustained 3-months after module completion, but this did not result in sustained behavior change. Implementation of effective education such as online learning modules in all medical education and health systems training programs may be one important step toward improving access to gender affirming care for youth.

## Electronic Supplementary Material

Below is the link to the electronic supplementary material.

Supplementary Material 1

## Data Availability

The datasets used and/or analysed during the current study are available from the corresponding author on reasonable request.

## References

[1] Olson J, Schrager SM, Belzer M, et al. Baseline Physiologic and Psychosocial Characteristics of Transgender Youth Seeking Care for Gender Dysphoria. J Adolesc Health. 2015;57(4):374–80. 10.1016/j.jadohealth.2015.04.027 26208863 10.1016/j.jadohealth.2015.04.027 PMC5033041

[2] Connolly MD, Zervos MJ, Barone CJ, et al. The Mental Health of Transgender Youth: Advances in Understanding. J Adolesc Health. 2016;59(5):489–95. 10.1016/j.jadohealth.2016.06.012 27544457 10.1016/j.jadohealth.2016.06.012

[3] Perez-Brumer A, Day JK, Russell ST, et al. Prevalence and Correlates of Suicidal Ideation Among Transgender Youth in California: Findings From a Representative, Population-Based Sample of High School Students. J Am Acad Child Adolesc Psychiatry. 2017;56(9):739–46. 10.1016/j.jaac.2017.06.010 28838578 10.1016/j.jaac.2017.06.010 PMC5695881

[4] Kuper LE, Mathews S, Lau M. Baseline Mental Health and Psychosocial Functioning of Transgender Adolescents Seeking Gender-Affirming Hormone Therapy. J Dev Behav Pediatr. 2019;40(8):589–96. 10.1097/DBP.0000000000000697 31166250 10.1097/DBP.0000000000000697

[5] Reisner SL, Vetters R, Leclerc M, et al. Mental health of transgender youth in care at an adolescent urban community health center: a matched retrospective cohort study. J Adolesc Health. 2015;56(3):274–9. 10.1016/j.jadohealth.2014.10.264 25577670 10.1016/j.jadohealth.2014.10.264 PMC4339405

[6] di Giacomo E, Krausz M, Colmegna F, et al. Estimating the Risk of Attempted Suicide Among Sexual Minority Youths: A Systematic Review and Meta-analysis. JAMA Pediatr. 2018;172(12):1145–52. 10.1001/jamapediatrics.2018.2731 30304350 10.1001/jamapediatrics.2018.2731 PMC6583682

[7] Corliss HL, Belzer M, Forbes C, et al. An evaluation of service utilization among male to female transgender youth: qualitative study of a clinic-based sample. J LGBT Health Res. 2007;3(2):49–61. 10.1300/J463v03n02_06 10.1300/J463v03n02_06 19835041

[8] Olson J, Forbes C, Belzer M. Management of the transgender adolescent. Arch Pediatr Adolesc Med. 2011;165(2):171–6. 10.1001/archpediatrics.2010.275 21300658 10.1001/archpediatrics.2010.275

[9] Wilson EC, Garofalo R, Harris RD, et al. Transgender female youth and sex work: HIV risk and a comparison of life factors related to engagement in sex work. AIDS Behav. 2009;13(5):902–13. 10.1007/s10461-008-9508-8 19199022 10.1007/s10461-008-9508-8 PMC2756328

[10] De Santis JP. HIV infection risk factors among male-to-female transgender persons: a review of the literature. J Assoc Nurses AIDS Care. 2009;20(5):362–72. 10.1016/j.jana.2009.06.005 19732695 10.1016/j.jana.2009.06.005

[11] de Vries ALC, McGuire JK, Steensma TD, et al. Young adult psychological outcome after puberty suppression and gender reassignment. Pediatrics. 2014;134(4):696–704. 10.1542/peds.2013-2958 25201798 10.1542/peds.2013-2958

[12] Turban JL, King D, Carswell JM, et al. Pubertal Suppression for Transgender Youth and Risk of Suicidal Ideation. Pediatrics. 2020;145(2):e20191725. 10.1542/peds.2019-1725 31974216 10.1542/peds.2019-1725 PMC7073269

[13] van der Miesen AIR, Steensma TD, de Vries ALC, et al. Psychological Functioning in Transgender Adolescents Before and After Gender-Affirmative Care Compared With Cisgender General Population Peers. J Adolesc Health. 2020;66(6):699–704. 10.1016/j.jadohealth.2019.12.018 32273193 10.1016/j.jadohealth.2019.12.018

[14] Fontanari AMV, Vilanova F, Schneider MA, et al. Gender Affirmation Is Associated with Transgender and Gender Nonbinary Youth Mental Health Improvement. LGBT Health. 2020;7(5):237–47. 10.1089/lgbt.2019.0046 32456545 10.1089/lgbt.2019.0046

[15] Tordoff DM, Wanta JW, Collin A, et al. Mental Health Outcomes in Transgender and Nonbinary Youths Receiving Gender-Affirming Care. JAMA Netw Open. 2022;5(2):e220978. 10.1001/jamanetworkopen.2022.0978 35212746 10.1001/jamanetworkopen.2022.0978 PMC8881768

[16] Allen LR, Watson LB, Egan AM, et al. Well-being and suicidality among transgender youth after gender-affirming hormones. Clin Pract Pediatr Psychol. 2019;7:302–11. 10.1037/cpp0000288

[17] Gridley SJ, Crouch JM, Evans Y, et al. Youth and Caregiver Perspectives on Barriers to Gender-Affirming Health Care for Transgender Youth. J Adolesc Health. 2016;59(3):254–61. 10.1016/j.jadohealth.2016.03.017 27235374 10.1016/j.jadohealth.2016.03.017

[18] Safer JD, Coleman E, Feldman J, et al. Barriers to healthcare for transgender individuals. Curr Opin Endocrinol Diabetes Obes. 2016;23(2):168–71. 10.1097/MED.0000000000000227 26910276 10.1097/MED.0000000000000227 PMC4802845

[19] Chong LSH, Kerklaan J, Clarke S, et al. Experiences and Perspectives of Transgender Youths in Accessing Health Care: A Systematic Review. JAMA Pediatr. 2021;175(11):1159–73. 10.1001/jamapediatrics.2021.2061 34279538 10.1001/jamapediatrics.2021.2061

[20] Weixel T, Wildman B. Geographic Distribution of Clinical Care for Transgender and Gender-Diverse Youth. Pediatrics. 2022;150(6):e2022057054. 10.1542/peds.2022-057054 36443242 10.1542/peds.2022-057054

[21] Korpaisarn S, Safer JD. Gaps in transgender medical education among healthcare providers: A major barrier to care for transgender persons. Rev Endocr Metab Disord. 2018;19(3):271–5. 10.1007/s11154-018-9452-5 29922962 10.1007/s11154-018-9452-5

[22] Vance SRJ, Halpern-Felsher BL, Rosenthal SM. Health care providers’ comfort with and barriers to care of transgender youth. J Adolesc Health. 2015;56(2):251–3. 10.1016/j.jadohealth.2014.11.002 25620310 10.1016/j.jadohealth.2014.11.002

[23] McPhail D, Rountree-James M, Whetter I. Addressing gaps in physician knowledge regarding transgender health and healthcare through medical education. Can Med Educ J. 2016;7(2):e70–8. 28344694 PMC5344057

[24] Unger CA. Care of the transgender patient: a survey of gynecologists’ current knowledge and practice. J Womens Health (Larchmt). 2015;24(2):114–8. 10.1089/jwh.2014.4918 25525682 10.1089/jwh.2014.4918

[25] Snelgrove JW, Jasudavisius AM, Rowe BW, et al. Completely out-at-sea with two-gender medicine: a qualitative analysis of physician-side barriers to providing healthcare for transgender patients. BMC Health Serv Res. 2012;12:110–110. 10.1186/1472-6963-12-110 22559234 10.1186/1472-6963-12-110 PMC3464167

[26] Hayes V, Blondeau W, Bing-You RG. Assessment of Medical Student and Resident/Fellow Knowledge, Comfort, and Training With Sexual History Taking in LGBTQ Patients. Fam Med. 2015;47(5):383–7. 25905882

[27] Johnston CD, Shearer LS. Internal Medicine Resident Attitudes, Prior Education, Comfort, and Knowledge Regarding Delivering Comprehensive Primary Care to Transgender Patients. Transgend Health. 2017;2(1):91–5. 10.1089/trgh.2017.0007 28861552 10.1089/trgh.2017.0007 PMC5548411

[28] Chen D, Berona J, Chan Y, et al. Psychosocial Functioning in Transgender Youth after 2 Years of Hormones. N Engl J Med. 2023;388(3):240–50. 10.1056/NEJMoa2206297 36652355 10.1056/NEJMoa2206297 PMC10081536

[29] Obedin-Maliver J, Goldsmith ES, Stewart L, et al. Lesbian, Gay, Bisexual, and Transgender–Related Content in Undergraduate Medical Education. JAMA. 2011;306(9):971–7. 10.1001/jama.2011.1255 21900137 10.1001/jama.2011.1255

[30] White W, Brenman S, Paradis E, et al. Lesbian, Gay, Bisexual, and Transgender Patient Care: Medical Students’ Preparedness and Comfort. Teach Learn Med. 2015;27(3):254–63. 10.1080/10401334.2015.1044656 26158327 10.1080/10401334.2015.1044656

[31] Pregnall AM, Churchwell AL, Ehrenfeld JM. A Call for LGBTQ Content in Graduate Medical Education Program Requirements. Acad Med. 2021;96(6):828–35. 10.1097/ACM.0000000000003581 34031304 10.1097/ACM.0000000000003581

[32] Coutin A, Wright S, Li C, et al. Missed opportunities: are residents prepared to care for transgender patients? A study of family medicine, psychiatry, endocrinology, and urology residents. Can Med Educ J. 2018;9(3):e41–55. 30140346 PMC6104317

[33] Morrison SD, Dy GW, Chong HJ, et al. Transgender-Related Education in Plastic Surgery and Urology Residency Programs. J Grad Med Educ. 2017;9(2):178–83. 10.4300/JGME-D-16-00417.1 28439350 10.4300/JGME-D-16-00417.1 PMC5398132

[34] Leyva VL, Breshears EM, Ringstad R. Assessing the efficacy of LGBT cultural competency training for aging services providers in California’s central valley. J Gerontol Soc Work. 2014;57(2–4):335–48. 10.1080/01634372.2013.872215 24341968 10.1080/01634372.2013.872215

[35] Dubin SN, Nolan IT, Streed CGJ, et al. Transgender health care: improving medical students’ and residents’ training and awareness. Adv Med Educ Pract. 2018;9:377–91. 10.2147/AMEP.S147183 29849472 10.2147/AMEP.S147183 PMC5967378

[36] Cooper RL, Ramesh A, Radix AE et al. Affirming and Inclusive Care Training for Medical Students and Residents to Reducing Health Disparities Experienced by Sexual and Gender Minorities: A Systematic Review. Transgender Health 2022 EPub; 10.1089/trgh.2021.0148 10.1089/trgh.2021.0148 PMC10387161 37525832

[37] Hana T, Butler K, Young LT, et al. Transgender health in medical education. Bull World Health Organ. 2021;99(4):296–303. 10.2471/BLT.19.249086 33953447 10.2471/BLT.19.249086 PMC8085635

[38] Nolan IT, Blasdel G, Dubin SN, et al. Current State of Transgender Medical Education in the United States and Canada: Update to a Scoping Review. J Med Educ Curric Dev. 2020;7. 10.1177/2382120520934813 10.1177/2382120520934813 PMC7315660 32637641

[39] Vance SRJ, Deutsch MB, Rosenthal SM, et al. Enhancing Pediatric Trainees’ and Students’ Knowledge in Providing Care to Transgender Youth. J Adolesc Health. 2017;60(4):425–30. 10.1016/j.jadohealth.2016.11.020 28065519 10.1016/j.jadohealth.2016.11.020

[40] Vance SRJ, Lasofsky B, Ozer E, et al. Teaching paediatric transgender care. Clin Teach. 2018;15(3):214–20. 10.1111/tct.12780 29573566 10.1111/tct.12780

[41] Oller D. Cancer Screening for Transgender Patients: An Online Case-Based Module. MedEdPORTAL. 2019;15:10796. 10.15766/mep_2374-8265.10796 30800996 10.15766/mep_2374-8265.10796 PMC6376892

[42] Cardea Training Center. Caring for Gender Diverse Youth. 2021. https://cardea.matrixlms.com/visitor_catalog_class/show/1251454 [Last accessed February 18, 2024].

[43] Coleman E, Radix AE, Bouman WP, et al. Standards of Care for the Health of Transgender and Gender Diverse People, Version 8. Int J Transgend Health. 2022;23(Suppl 1):S1–259. 10.1080/26895269.2022.2100644 36238954 10.1080/26895269.2022.2100644 PMC9553112

[44] Hembree WC, Cohen-Kettenis PT, Gooren L, et al. Endocrine Treatment of Gender-Dysphoric/Gender-Incongruent Persons: An Endocrine Society Clinical Practice Guideline. J Clin Endocrinol Metab. 2017;102(11):3869–903. 10.1210/jc.2017-01658 28945902 10.1210/jc.2017-01658

[45] UCSF Transgender Care, Department of Family and Community Medicine, University of California San Francisco. Guidelines for the Primary and Gender affirming Care of Transgender and Gender Nonbinary People. 2016; 2nd edition. Deutsch MB, ed. transcare.ucsf.edu/guidelines [Last accessed June 26, 2023].

[46] Kattari SK, Curley KM, Bakko M, et al. Development and Validation of the Trans-Inclusive Provider Scale. Am J Prev Med. 2020;58(5):707–14. 10.1016/j.amepre.2019.12.005 32044143 10.1016/j.amepre.2019.12.005

[47] Hodax JK, Kahn NF, Crouch JM, Bocek KM, Goldenberg M, Loren D, Sethness JL, Sumerwell C, Sequeira GM. A Project ECHO improves provider confidence and behaviors in gender-affirming care for youth. Transgender Health. In. 10.1089/trgh.2023.0171 PMC12670656 41340814

[48] StataCorp. Stata Statistical Software: Release 14. StataCorp LP; 2015.

[49] American Civil Liberties Union. Mapping Attacks on LGBTQ Rights in the U.S. State Legislatures. ACLU: New York, NY. 2023. https://www.aclu.org/legislative-attacks-on-lgbtq-rights?impact=health [Last accessed May 22, 2023].

[50] Streed CGJ, Navarra M, Klein J. Advancing undergraduate medical education regarding the care of transgender and gender Diverse persons and communities. Perspect Med Educ. 2022;11(6):306–8. 10.1007/s40037-022-00732-w 36435909 10.1007/s40037-022-00732-w PMC9743931

[51] Olson KR, Durwood L, DeMeules M, et al. Mental Health of Transgender Children Who Are Supported in Their Identities. Pediatrics. 2016;137(3):e20153223. 10.1542/peds.2015-3223 26921285 10.1542/peds.2015-3223 PMC4771131

[52] Russell ST, Pollitt AM, Li G, et al. Chosen Name Use Is Linked to Reduced Depressive Symptoms, Suicidal Ideation, and Suicidal Behavior Among Transgender Youth. J Adolesc Health. 2018;63(4):503–5. 10.1016/j.jadohealth.2018.02.003 29609917 10.1016/j.jadohealth.2018.02.003 PMC6165713

