# Supplementary material for: Online learning modules improve confidence in providing gender affirming care for youth
Source: BMC Med Educ. 2024 Dec 19;24:1498. doi: 10.1186/s12909-024-06517-5 (PMC11660828; doi:10.1186/s12909-024-06517-5)
Supplement: Supplementary file 1 — Supplementary Material 1 [file 12909_2024_6517_MOESM1_ESM.docx]

**Appendix 1. Online Module Evaluation Survey**

**Demographics**

1. What type of provider are you?
   1. Physician (MD, DO)
   2. Naturopathic medicine provider (ND)
   3. Advanced practice provider (ARNP, PA)
   4. Resident (MD, DO)
   5. Medical student or health professional trainee
   6. Mental health provider (psychology, social work)
   7. Nurse
   8. Medical assistant
   9. Other ___
2. Not including your time in training, how many years have you been in practice?

____

1. What zip-code is your primary clinic located in?

____

1. Do your patients have access to a mental health provider within your clinic?
2. Yes
3. No
4. Other:___
5. In your best estimate, what % of patients on your panel have medicaid insurance?
6. 76-100%
7. 51-75%
8. 26-50%
9. Less than 25%
10. How many gender diverse patients under 18 have you seen in your practice?
11. 5 or less
12. 6-10
13. 11-15
14. More than 15

**Module 1**

**Section 1**- (Response options: Yes, No, Not sure)

**Intake forms**

1. My practice’s intake forms have a space for patients to share their pronouns
2. My practice’s intake forms have a space for patients to share their affirmed name
3. My practice’s intake forms have multiple gender options and/or a blank for patients to fill in their gender
4. My practice’s EHR allows me to include a name different than a patient’s legal name
5. My practice’s EHR allows me to include a patient’s pronouns

**Internalized trans-inclusive knowledge and actions**

1. My pronouns are currently visible to others (ID badge, email signature, Zoom name, etc)
2. My practice has a gender-inclusive bathroom that patients can access
3. My office staff has taken trainings specifically on transgender 101 topics (such as what pronouns signify and what transgender identities might be)
4. My practice employs transgender or nonbinary staff members
5. I have taken trainings specifically on transgender health topics
6. I have done my own research on best practices for trans health care

**Passive trans-inclusive marketing**

1. My practice’s website contains a rainbow flag, transgender pride flag or the words *transgender, trans health, or trans(gender) inclusive*
2. My practice’s printed materials contain a rainbow pride flag or transgender pride flag/the words *transgender, trans health, or trans(gender) inclusive*
3. My practice has a rainbow pride flag or transgender pride flag on the office door or in the waiting room

**Section 2-** (Response options: 1 – This is not true at all, 2 – This is rarely true, 3 – This is somewhat true, 4 – This is usually true, 5 – This is always true)

**Provider behaviors**

1. Other providers in my practice use patients’ affirmed name and pronouns
2. My office staff use patients’ affirmed name and pronouns
3. I introduce myself to patients with my name and pronouns
4. I ask my patients what name and pronouns they use during every clinical encounter
5. I use my patient’s affirmed name and pronouns when speaking with them
6. I use my patients’ affirmed name and pronouns when describing them to others
7. I initiate conversations about gender identity with patients during every routine visit
8. With their consent, I include my patients affirmed name and pronouns in the EHR
9. With their consent, I include my patients gender identity in the EHR

**Provider confidence**

**I am confident in my ability to...**

1. introduce myself to patients with my name and pronouns
2. initiate conversations about gender identity with patients during routine visits
3. support a gender diverse patient in talking with their parent or caregiver about their gender identity
4. explain to a patient or family the difference between gender identity and sexual orientation
5. explain to a parent or caregiver who is refusing to use a gender diverse patient's name and pronouns the negative impact this has on mental health (with patient’s consent)
6. explain to a teacher or school administrator the importance of using a gender diverse patient's name and pronouns at school (with patient’s consent)
7. provide information to a patient about local, gender-affirming resources youth can access confidentially (ie. without parental involvement)
8. provide information to a patient or family about local, gender-affirming resources or supports for parents or caregivers of gender diverse youth

**Module 2**

**Section 1-** (Response options: 1 – This is not true at all, 2 – This is rarely true, 3 – This is somewhat true, 4 – This is usually true, 5 – This is always true)

**Provider confidence**

**I am confident in my ability to...**

1. counsel patients and families about puberty blockers (such as what they do, when they can be initiated, how they are administered, etc)
2. counsel patients and families about hormone therapy like Testosterone and Estradiol (such as what it does, when it can be initiated, how it can be administered, etc)
3. refer a gender diverse young person under 18 to a local provider who prescribes puberty blockers and gender affirming hormones
4. refer a gender diverse young person under 18 for a gender-affirming surgery

**Section 2-** **for prescribers only**

(Response options: 1 – This is not true at all, 2 – This is rarely true, 3 – This is somewhat true, 4 – This is usually true, 5 – This is always true)

**I am confident in my ability to...**

1. determine pubertal stage for a patient who is interested in receiving puberty blockers
2. prescribe medications to suppress menses for gender diverse youth experiencing dysphoria with bleeding
3. prescribe an initial prescription for puberty blockers
4. prescribe a follow up prescription for puberty blockers after another provider prescribed the initial prescription
5. perform lab monitoring for patients receiving puberty blockers
6. prescribe an initial prescription for gender-affirming hormones (Testosterone, Estradiol)
7. prescribe a follow up prescription for gender-affirming hormones (Testosterone, Estradiol) for a patient under age 18 after another provider prescribed the first prescription
8. perform lab monitoring for patients receiving gender-affirming hormones (Testosterone, Estradiol)
